# Radiomics to discriminate between axial spondyloarthritis and axial psoriatic arthritis and to predict TNFi therapy persistence

**DOI:** 10.1016/j.ero.2025.12.007

**Published:** 2025-12-27

**Authors:** Vincenzo Venerito, Sergio Del Vescovo, Crescenzio Scioscia, Maria Giannotta, Michele De Ceglie, Antonio Vitale, Florenzo Iannone, Giuseppe Lopalco

**Affiliations:** 1 Rheumatology Unit–Department of Precision and Regenerative Medicine and Ionian Area, University of Bari, Bari, Italy; 2 Radiology Unit–AOCU Policlinico di Bari, Bari, Italy; 3 Department of Medical Sciences, Surgery and Neurosciences, Research Center of Systemic Autoinflammatory Diseases and Behçet’s Disease Clinic, University of Siena, Siena, Italy

## Abstract

**Objectives:**

Axial spondyloarthritis (axSpA) and axial psoriatic arthritis (axPsA) represent entities with ongoing debate regarding their classification as distinct diseases or variants of the same condition. Although magnetic resonance imaging (MRI) is instrumental in diagnosing, traditional assessment may not fully capture subtle differences between them. This study aims to investigate whether radiomic features extracted from sacroiliac joint (SIJ) bone marrow oedema (BME) on MRI can discriminate between axSpA and axPsA, and predict tumour necrosis factor inhibitor (TNFi) therapy persistence in patients with axSpA.

**Methods:**

We included patients who underwent an SIJ MRI at our hospital. BME regions were segmented on short-tau inversion-recovery sequences, and 120 standardised radiomic features were extracted using PyRadiomics. For diagnostic discrimination, an XGBoost algorithm was employed and evaluated through 5-fold cross-validation. For patients with axSpA who initiated TNFi therapy, an exploratory Cox regression analysis was performed to identify radiomic predictors of treatment persistence.

**Results:**

We analysed MRI scans from 66 patients (41 axSpA, 25 axPsA). The XGBoost classifier achieved an accuracy of 0.80 ± 0.07 and area under the receiver operating characteristic curve of 0.77 ± 0.09 in differentiating axSpA from axPsA. The most discriminative features included texture parameters, shape characteristics, and grey-level intensity distributions. For TNFi persistence, multivariate Cox regression identified 2 shape-based features as independent predictors: increased sphericity, associated with discontinuation risk (hazard ratio [HR] 2.10, 95% CI: 1.09-4.05), while increased elongation showed a protective effect (HR 0.50, 95% CI: 0.25-0.99).

**Conclusions:**

This proof-of-concept study suggests radiomic SIJ BME features may help differentiate axSpA and axPsA and could be associated with TNFi persistence in patients with axSpA. These preliminary findings suggest that these conditions may exhibit distinct radiomic phenotypes.

**WHAT IS ALREADY KNOWN**

• Axial spondyloarthritis and axial psoriatic arthritis have overlapping clinical presentations, and traditional MRI assessment may not capture subtle differences between these conditions.

**WHAT THIS STUDY ADDS**

• Radiomic features from the sacroiliac joint bone marrow oedema may discriminate between axial spondyloarthritis and axial psoriatic arthritis and may be associated with TNF inhibitors persistence.

**HOW THIS STUDY MIGHT AFFECT RESEARCH, PRACTICE OR POLICY**

• Radiomics may serve as a non-invasive tool to support differential diagnosis and treatment decisions, though validation in larger multicentre cohorts is needed before clinical implementation.

Alt-text: Unlabelled box dummy alt text

## INTRODUCTION

Axial spondyloarthritis (axSpA) and axial psoriatic arthritis (axPsA) represent significant challenges in rheumatology due to their usually overlapping clinical presentations [1]. This overlap has sparked considerable debate within the rheumatology community, giving rise to 2 distinct schools of thought: the ‘lumpers’, who advocate for grouping these conditions under a broader category, and the ‘splitters’, who argue that they represent distinct entities [2].

The distinction between axSpA and axPsA has been a subject of ongoing debate because the first recognition of axial involvement in psoriatic arthritis (PsA) 50 years ago [[3], [4], [5], [6]]. Recent evidence suggests fundamental differences in their pathophysiology, particularly in terms of tissue involvement. AxSpA, in fact, may be characterised by predominant bone involvement with perifibrocartilaginous sacroiliac joint (SIJ) osteitis, whereas axPsA may manifest primarily as inflammation in ligamentous soft tissue or ‘ligamentitis’, with distinctive features including para-syndesmophytes and SIJ bone sparing [7,8].

Magnetic resonance imaging (MRI) has become instrumental in the diagnosis and monitoring of both conditions, with bone marrow oedema (BME) of the SIJs serving as a key imaging finding [9,10]. However, its traditional qualitative assessment of imaging features may not fully capture the subtle differences between these conditions. In this regard, in recent years, radiomics has emerged as a powerful tool in medical imaging analysis, particularly in oncology, where it allows thorough phenotype quantification through detailed examination of medical images’ characteristics [11]. Through specialised software, it becomes possible to segment regions of interest (ROI) on MRI images and extract numerous features that may reveal disease characteristics not directly apparent to the human eye. The fundamental hypothesis of radiomics suggests that distinctive imaging features between disease forms may aid in diagnosis and also to predict therapeutic response, thereby providing valuable information for personalised therapy and patient management. Our study objective was to investigate whether features from radiomic analysis of SIJ BME could effectively discriminate between axSpA and axPsA, while also exploring their potential to predict tumour necrosis factor inhibitor (TNFi) persistence in patients with spondyloarthritis.

## METHODS

### Patient selection and clinical assessment

We conducted a comprehensive analysis of MRI SIJ scans from patients with confirmed diagnoses of axSpA and axPsA who were followed at our Rheumatology department between January 2017 and December 2023. Patients with AxSpA were classified according to the Assessment of SpondyloArthritis international Society criteria for axSpA [9], while patients with axPsA fulfilled the ClASsification criteria for Psoriatic Arthritis [12] and additionally demonstrated axial involvement on SIJ MRI. Importantly, for standardisation purposes in this study, in order to be enrolled in the axSpA group, patients were required to not have Psoriasis (PsO) or any peripheral joint involvement. For each patient, we carefully collected detailed clinical and demographic characteristics, including age at MRI, body mass index (BMI), disease duration, C-reactive protein (CRP), *Human Leukocyte Antigen-B27* (*HLA-B27*) status, and Axial Spondyloarthritis Disease Activity Score-CRP (ASDAS-CRP) scores.

For the second part of the study, focusing on TNFi therapy persistence, we included those patients with axSpA classified according to the same criteria described above, who initiated TNFi therapy after their baseline SIJ MRI examination; and then, we tracked the time to eventual treatment discontinuation, with discontinuation defined as permanent cessation of the TNFi agent for any reason. Also, for this group of patients, we collected clinical and demographic characteristics, including sex, age at MRI, BMI, disease duration, CRP, *HLA-B27* status, ASDAS-CRP, line of therapy, and specific TNFi prescribed.

For inclusion in such retrospective analysis, all MRI scans needed to be performed at our institution’s Radiology department, with Digital Imaging and Communication in Medicine (DICOM) files stored in our picture archiving and communication system.

We excluded patients whose MRI scans failed to meet our quality standards (see below) or had incomplete sequences or incomplete BME representation. The study was approved and reviewed by the local Ethical Committee (Biopure registry, IRP Approval no. 5940, Azienda Ospedaliera Universitaria di Bari). All patients gave their written informed consent.

### Image acquisition, processing and radiomics analysis

All MRI examinations were performed on a Philips Achieva Nova Dual 1.5 T scanner (Philips Healthcare) using a dedicated spine coil and a standardised protocol that included axial short-tau inversion-recovery (STIR) sequences of the SIJs.

**Figure 1:**
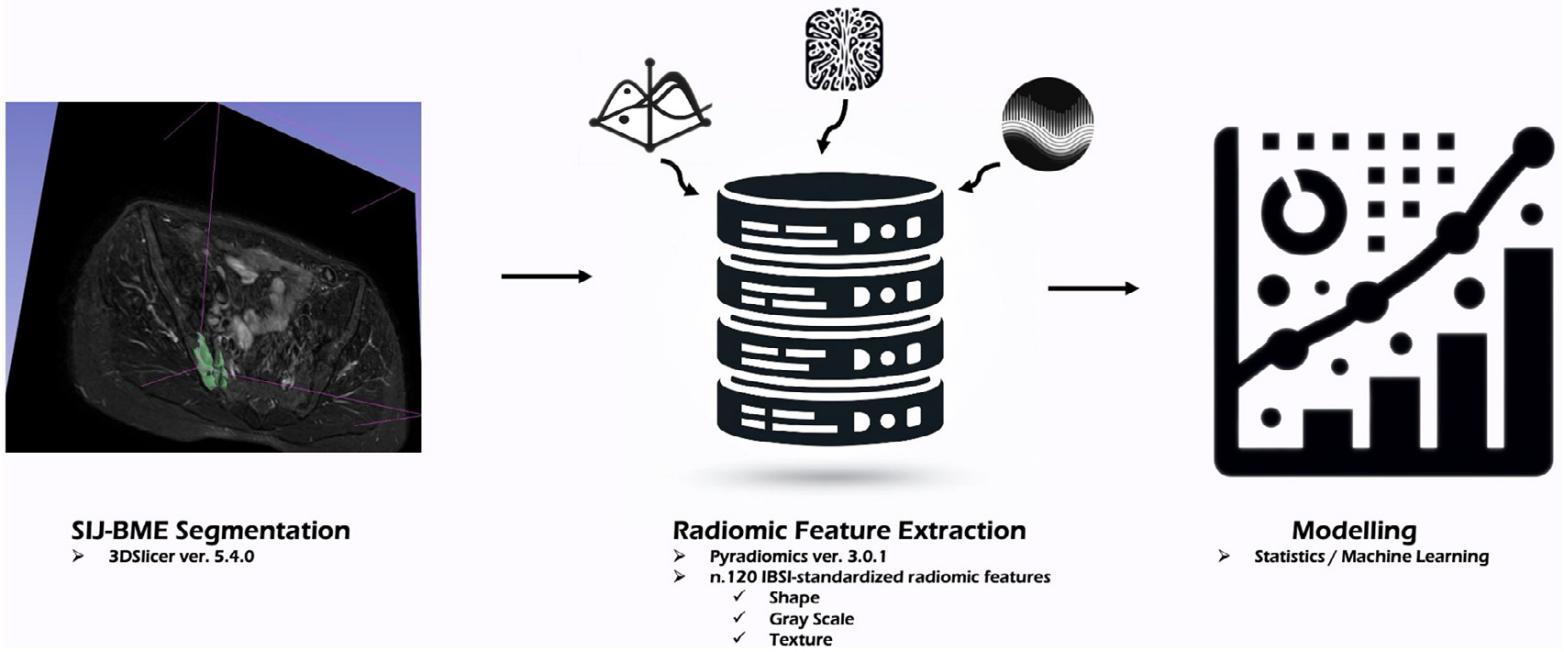
Radiomic workflow for sacroiliac joint (SIJ) bone marrow oedema (BME) analysis. The pipeline consists of 3 main steps: (1) semiautomated segmentation of bone marrow oedema regions on STIR sequences using 3D Slicer software version 5.4.0; (2) extraction of 120 IBSI-standardised radiomic features using PyRadiomics version 3.0.1; (3) statistical analysis and machine learning modelling using XGBoost algorithm for classification and Cox regression for survival analysis. IBSI, image biomarker standardisation initiative; STIR, short-tau inversion-recovery; 3D, 3-dimensional.

Our volumetric analysis and visualisation were performed using 3-dimensional (3D) Slicer software ver 5.4.0 [13]. An expert radiologist with 10 years of experience in musculoskeletal imaging performed semiautomatic segmentation of the BME on STIR sequences (Fig 1, Supplementary Video) using the ‘Level Tracing’ function within the Segment Editor module of the software [13].

The PyRadiomics platform was developed for cancer research with the US National Cancer Institute grant 5U24CA194354 [14]. It can extract radiomic data from medical imaging loading, preprocess the image and segmentation maps, calculate features using the different feature classes, and return results as continuous variables in Comma-Separated Values (CSV) format. We implemented the PyRadiomics platform (version 3.0.1) within a Python 3.9 environment to extract features from the segmented regions. All radiomic features were extracted using PyRadiomics version 3.0.1 following image biomarker standardisation initiative (IBSI) guidelines specifically adapted for MRI analysis.

All STIR images were resampled to isotropic 1 × 1 × 1 mm voxel spacing using B-spline interpolation to ensure spatial consistency across all cases and enable reliable texture analysis. Given the arbitrary intensity units inherent to MRI, we applied fixed bin count discretisation using 64 discrete grey levels, as recommended by IBSI for nonquantitative imaging modalities. This approach ensures consistent texture quantisation regardless of individual image intensity ranges.

Images were padded with a 5-voxel border to minimise edge effects during texture calculation, and all preprocessing maintained the original image geometry and orientation.

From each segmented BME region, we extracted 120 standardised radiomic features across 7 distinct feature classes, each capturing different aspects of tissue characteristics (see Supplementary Material for a description of these 7 classes).

### Sample size justification

For the diagnostic classification model of axSpA vs axPsA, we followed established radiomics guidelines requiring 10 to 15 patients per feature for stable machine learning performance [15]. We targeted a core set of 6 radiomic features, necessitating a minimum of 60 to 90 patients. Our achieved sample of 66 patients provides an 11:1 patient-to-feature ratio, which meets the recommended threshold and falls within the acceptable range for preventing overfitting in high-dimensional medical imaging data.

For survival analysis of patients on TNFi, we initially targeted a 3-predictor Cox regression model based on established guidelines requiring 10 to 15 events per variable [16]. This would have required 30 to 45 events to maintain adequate statistical power. However, our TNFi cohort of 32 patients yielded only 10 observed discontinuation events (31% event rate), providing sufficient power for a maximum of 2 predictors while maintaining the minimum 5 events-per-variable threshold for stable coefficient estimation.

### Segmentation quality control

To ensure segmentation validity in our single-operator design, we implemented a systematic quality control protocol:

Each segmented BME region was checked for reasonable volume ranges. Segmentations with unusually small (<100 mm³) or large (>10,000 mm³) volumes were visually reviewed to confirm anatomical accuracy. All segmented regions were verified to be located within the SIJ subchondral bone marrow, excluding inadvertent inclusion of adjacent soft tissues or cortical bone. Segmented regions were confirmed to display appropriate hyperintense signal on STIR sequences consistent with BME, excluding areas of artefact or non-BME pathology. To evaluate segmentation consistency, 15 randomly selected cases (23% of the total cohort) were resegmented by the same operator after a 2-week interval. This achieved excellent reliability with Dice coefficients >0.90 and intraclass correlation coefficients (ICC) of 0.95 for radiomic features.

### Feature reduction and correlation filtering

Before model training, we reduced feature dimensionality through correlation analysis to avoid redundancy and mitigate multicollinearity. Features showing high intercorrelation (Pearson |ρ| > 0.8) were considered collinear, and only 1 representative feature from each correlated cluster was retained based on biological plausibility and stability across folds. This filtering step yielded 39 independent radiomic variables for subsequent analysis (see Supplementary Fig).

### Statistical and machine learning analysis

To reduce complexity and mitigate overfitting, we implemented recursive feature elimination (RFE) using an extreme gradient boosting’s (XGBoost’s) built-in feature importance as the ranking criterion. RFE was nested within each training fold of the 5-fold cross-validation (CV) to prevent information leakage. In each iteration, the model was retrained with progressively fewer features until the top 6 most predictive radiomic variables were retained. The final model was trained using these 6 features and evaluated within the same nested CV scheme.

For classification of axSpA vs axPsA, we employed a new XGBoost algorithm using only radiomic features, without incorporating any clinical parameters. XGBoost is an ensemble machine learning method that combines multiple decision trees sequentially, where each new tree learns to correct errors from previous trees, resulting in improved predictive accuracy through gradient descent optimisation. The model was evaluated with 5-fold cross-validation, reporting its performance by the mean area under the receiver operating characteristic curve (AUROC). Within each outer-fold training set, we performed a grid-search for hyperparameters (Supplementary Table S1), and XGBoost training employed early-stopping that monitored mean AUROC on a 15% validation split of that same training data; training was halted if AUROC failed to improve by ≥0.001 for 25 consecutive boosting rounds (maximum 500 trees). Both the hyperparameter search and the early-stopping procedure were therefore fully nested inside the outer cross-validation loop, eliminating any risk of optimistic bias.

In order to analyse TNFi therapy persistence, a univariate Cox regression analysis was first conducted to identify potential predictors, considering variables with *P* < .25 as candidates. Then, a multivariate Cox regression model was constructed through backward selection, retaining variables only with *P* < .05. The model’s reliability was assessed by plotting Cox-Snell residuals against the Nelson-Aalen cumulative hazard rate.

No imputation for missing data was necessary.

Stata17 (StataCorp), together with Python 3.9, herein invoked with Pystata API, numpy 1.22, pandas 1.4.3, and scikit-learn 1.1.2 libraries, was used on a terminal powered by an Apple Silicon M1Max with 64 GB RAM. The study adheres to the TRIPOD+AI checklist [17].

## RESULTS

The first part of our study included a total of 66 patients (Table 1), with 41 patients (62.12%) diagnosed with axSpA and 25 patients (37.87%) with axPsA. In the axSpA cohort, a predominance of male patients was observed, comprising 28 individuals (68.29%) with a mean age of 41.09 ± 15.27 years. The axPsA group showed similar demographics, including 14 male patients (56.00%) and a mean age of 45.01 ± 11.73 years. Disease activity, as measured by ASDAS-CRP, was comparable between the groups, with patients with axSpA showing a mean score of 2.69 ± 0.97 and patients with axPsA 2.78 ± 0.80.

**Table 1:** Disease features of patients recruited with axSpA and axPsA

Features	AxSpA (n = 41)	AxPsA (n = 25)
Male, n (%)	28 (68.29)	14 (56.00)
Age, mean (SD)	41.09 (15.27)	45.08 (11.73)
BMI, mean (SD)	25 (4.35)	28.49 (6.60)
Disease duration in mo, mean (SD)	69.85 (68.63)	74.16 (66.69)
*HLA-B27* status, n (%)	20 (48.78)	1 (4.00)
CRP (mg/dL), mean (SD)	0.84 (1.43)	0.78 (1.03)
ASDAS-CRP, mean (SD)	2.70 (0.97)	2.87 (0.80)
Peripheral joint involvement, n (%)	0 (0)	25 (100.00)
Oligoarticular peripheral involvement, n (%)	0 (0)	20 (80.00)
Personal PsO, n (%)	0 (0)	25 (100.00)

ASDAS-CRP, Axial Spondyloarthritis Disease Activity Score-CRP; axPsA, axial psoriatic arthritis; axSpA, axial spondyloarthritis; BMI, body mass index; CRP, C-reactive protein; *HLA-B27, Human Leukocyte Antigen-B27*; PsO, Psoriasis.

*HLA-B27* positivity was substantially more prevalent in the axSpA group, present in 48.80% of patients compared to only 4.00% in the axPsA group (*P* < .001). We also found that BMI values were generally higher in the axPsA cohort (28.49 ± 6.6) compared to the axSpA group (25.0 ± 4.35) (*P* < .05). Disease duration was similar between groups, with patients with axSpA averaging 69.85 ± 68.63 months and patients with axPsA 74.16 ± 66.69 months. Inflammatory markers, specifically CRP levels, showed no difference between groups, with patients with axSpA averaging 0.84 ± 1.43 mg/dL and patients with axPsA 0.78 ± 1.03 mg/dL. Of note, 4 (9.76%) patients among the axSpA group and 2 (8%) patients in the axPsA group had prior exposure to TNFi, while the others were biologic-naive.

In our radiomic analysis, we initially extracted 120 standardised features from the segmented BME regions. After conducting correlation analysis and eliminating features with high collinearity (correlation coefficient > 0.8), 39 independent features were then retained for further analysis (Supplementary Fig).

**Figure 2:**
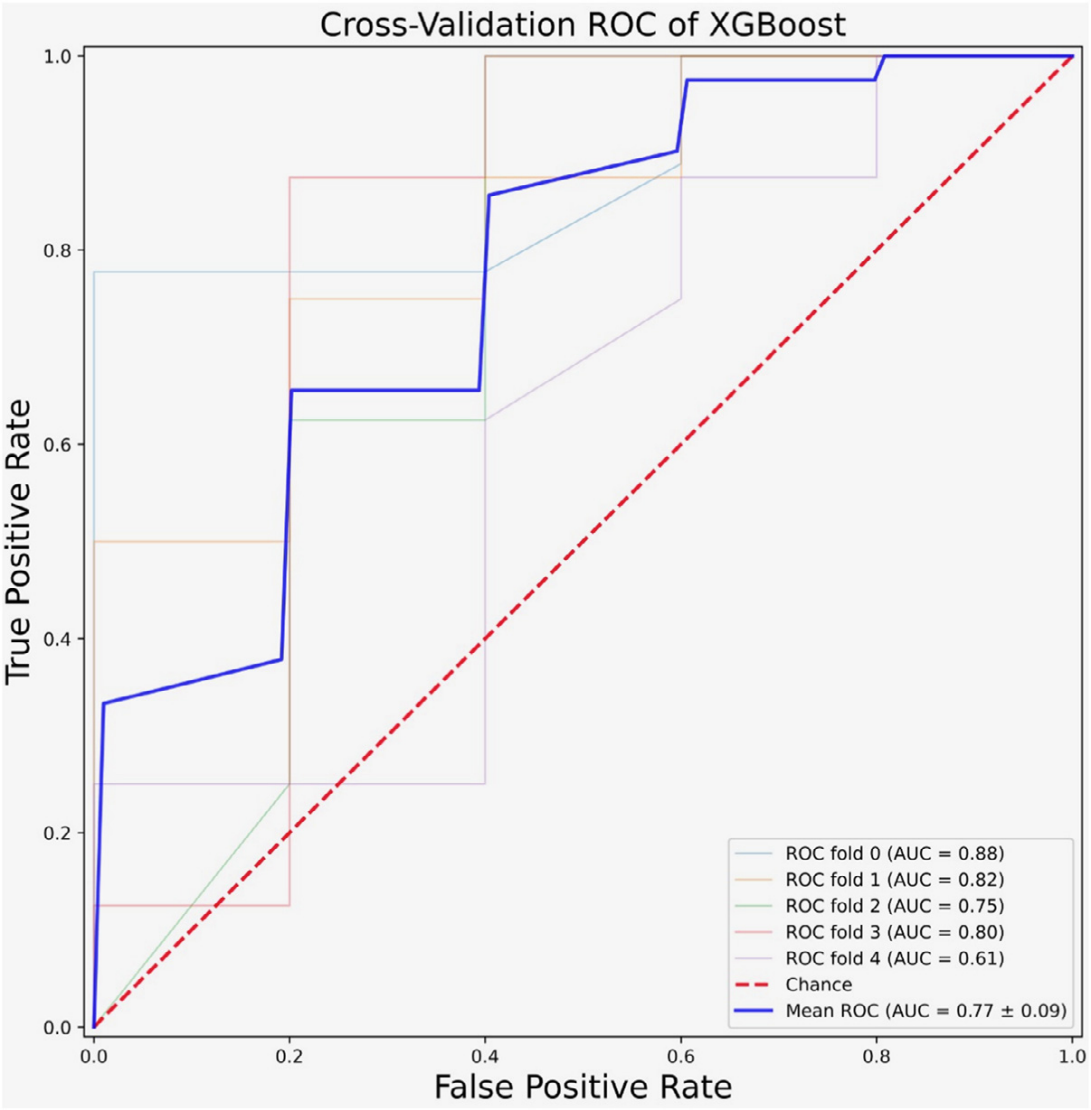
Cross-validation receiver operating characteristic (ROC) curves for XGBoost classifier. The figure displays the ROC curves from 5-fold cross-validation for discriminating axSpA from axPsA using radiomic features. The mean ROC curve (blue bold line) achieved an AUC of 0.77 ± 0.09. The red-dashed line represents chance performance (AUC = 0.50). axPsA, axial psoriatic arthritis; axSpA, axial spondyloarthritis; AUC, Area Under the Curve.

Then, the XGBoost classifier showed discrete performance in discriminating between axSpA and axPsA (Fig 2). Through 5-fold cross-validation, we achieved an accuracy of mean 0.80 ± SD 0.07, precision of 0.86 ± 0.10, recall of 0.86 ± 0.10, and an AUROC of 0.77 ± 0.09. Our RFE analysis identified 6 key radiomic characteristics that contributed most significantly to the diagnostic model.

**Figure 3:**
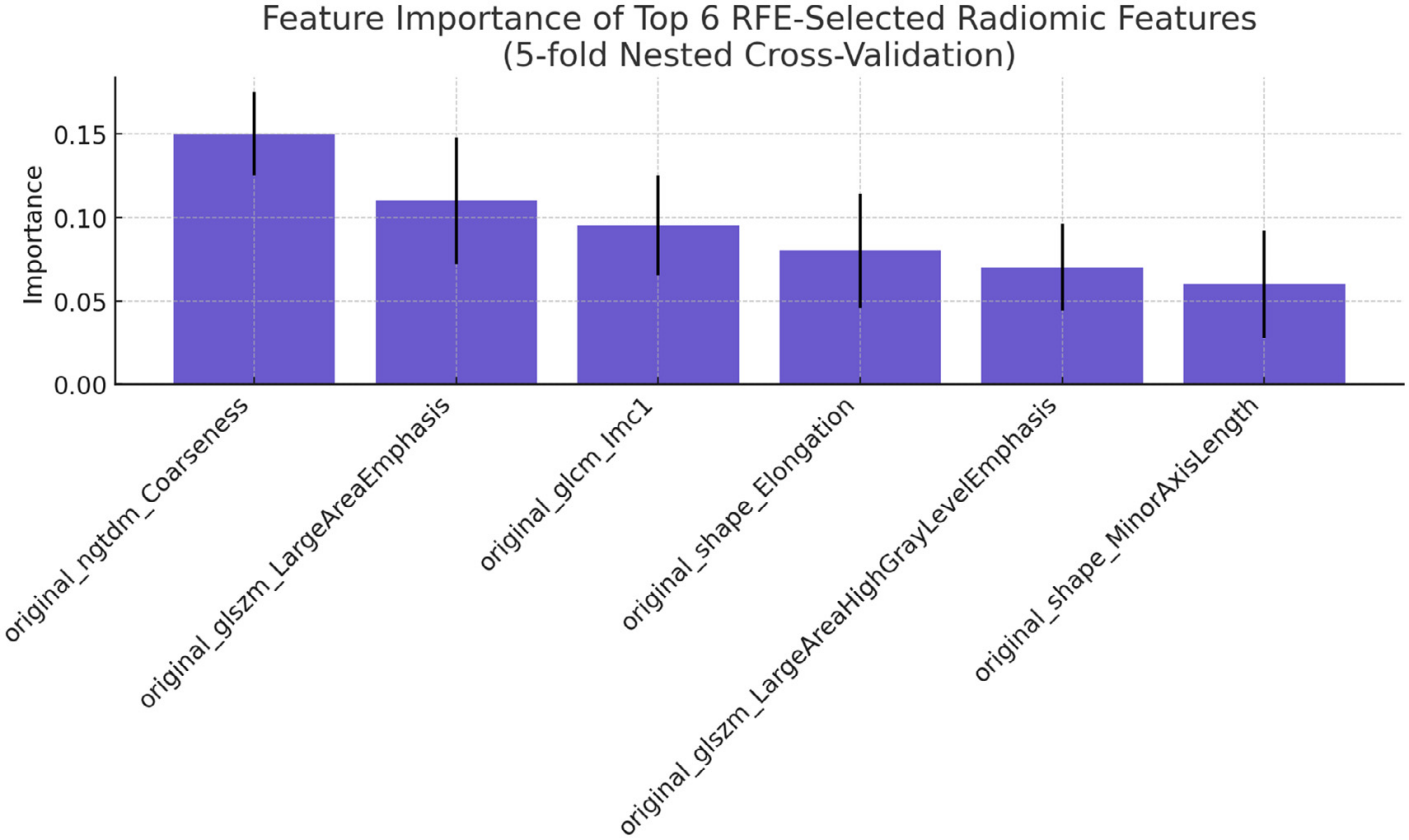
Feature importance of the top 6 radiomic features selected through recursive feature elimination. The bar plot shows the relative importance (mean ± SD across 5-fold nested cross-validation) of the 6 most discriminative radiomic features for differentiating axSpA from axPsA. Features include texture parameters (NGTDM Coarseness, GLCM Imc1), shape characteristics (Elongation, MinorAxisLength), and grey-level intensity distributions (GLSZM LargeAreaEmphasis, GLSZM LargeAreaHighGrayLevelEmphasis). axPsA, axial psoriatic arthritis; axSpA, axial spondyloarthritis; GLSZM, Gray Level Size Zone Metrics; GLCM, Gray Level Co-occurrence Matrix; IMC1, Informational Measure of Correlation 1; NGTDM, Neighboring Gray Tone Difference Matrix; RFE, recursive feature elimination.

Hence, the most important features for prediction (Fig 3) were components of:

• The texture of the BME: (*original_ngtdm_Coarseness, original_glcm_Imc1*) Neighboring Gray Tone Difference Matrix (NGTDM) Coarseness quantifies the spatial rate of change in intensity values, with higher values indicating more uniform local textures and lower spatial change rates in the BME patterns. Gray Level Co-occurrence Matrix (GLCM) Informational Measure of Correlation 1 (IMC1) assesses the correlation between probability distributions of intensity values, effectively measuring the complexity of the BME texture patterns through mutual information analysis.
• The shape of the BME (*original_shape_Elongation, original_shape_MinorAxisLength*); elongation, defined as the ratio of the principal component axes, quantifies the relationship between the 2 largest principal components in the ROI shape, ranging from 0 (maximally elongated) to 1 (perfectly circular cross-section). MinorAxisLength represents the second-largest axis length of the ROI-enclosing ellipsoid, offering insights into the dimensional extent of the lesions.
• The grey-level intensities of the BME (*original_glszm_LargeAreaEmphasis, original_glszm_LargeAreaHighGrayLevelEmphasis*). These features evaluate the distribution of large homogeneous zones within the BME regions, with LargeAreaEmphasis measuring the presence of large uniform areas and LargeAreaHighGrayLevelEmphasis specifically quantifying the presence of large, high-intensity regions.

On the other hand, in our analysis of TNFi therapy persistence, we focused on 32 patients with axSpA who received this treatment and had undergone an MRI before starting the therapy (Table 2). This group comprised 13 female patients (40.63%) with a mean age of 38.47 ± 13.08 years. The majority of patients (28, representing 87.50%) received TNFi as first-line therapy, while 4 patients (12.50%) had prior TNFi exposure. The median follow-up time was 17 months (IQR: 8.25-36). During the observation period, 10 discontinuation events (31.20%) were recorded, while 22 patients (68.80%) remained on therapy at the end of follow-up. Reasons of discontinuation included primary inefficacy (n = 5, 50.00%) and secondary inefficacy (n = 5, 50.00%), while no discontinuation was due to adverse events or other reasons in this cohort. The median drug survival time was 45 months (IQR: 18-not reached).

**Table 2:** Disease features of patients with axSpA who were prescribed TNFi after the MRI

Characteristic	axSpA (n = 32)
Female*, n* (%)	13 (40.60)
Age, y, mean (SD)	38.47 (13.08)
BMI*, mean (SD)*	25.59 (4.62)
Disease duration, mo*, mean (SD)*	67.97 (64.60)
*HLA-B27* positive, *n* (%)	14 (43.75)
CRP (mg/dL), mean (SD)	0.86 (1.30)
ASDAS-CRP, mean (SD)	2.55 (1.16)
1st line TNFi, *n* (%)	28 (87.50)
2nd line TNFi, *n* (%)	4 (12.50)
TNFi agents, *n* (%)	
Adalimumab	16 (50.00)
Etanercept	8 (25.00)
Certolizumab pegol	5 (15.63)
Golimumab	2 (6.25)
Infliximab	1 (3.13)
Treatment discontinuation, *n* (%)	10 (31.20)
Primary inefficacy	5 (50.00%)
Secondary inefficacy	5 (50.00%)
Adverse events	0
Drug survival time, mo, median (IQR)	45 (18-NR)

ASDAS-CRP, Axial Spondyloarthritis Disease Activity Score-CRP; axSpA, axial spondyloarthritis; BMI, body mass index; CRP, C-reactive protein; MRI, magnetic resonance imaging; NR, not reached; TNFi, tumour necrosis factor inhibitor; *HLA-B27, Human Leucocyte Antigen - B27.*

**Figure 4:**
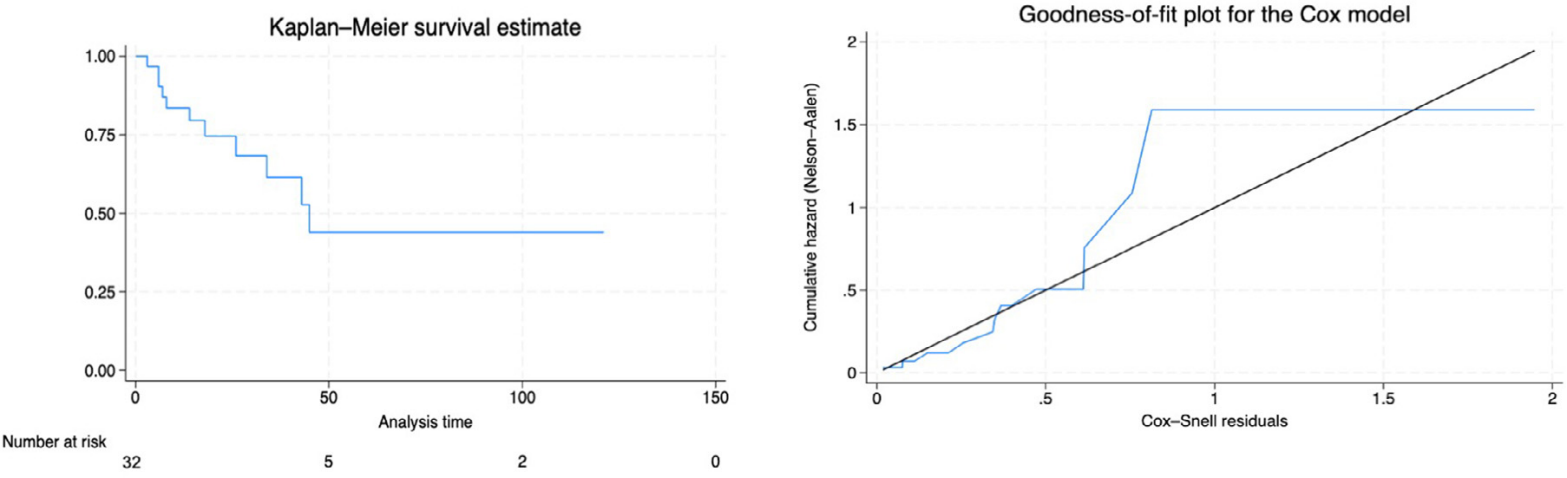
Survival analysis for TNFi therapy persistence in patients with axSpA. Left panel: Kaplan-Meier survival curve showing the probability of TNFi therapy continuation over time (n = 32 patients). Right panel: goodness-of-fit plot for the Cox proportional hazards model, showing Cox-Snell residuals plotted against Nelson-Aalen cumulative hazard. The alignment with the diagonal reference line indicates adequate model fit, particularly during earlier time periods where most discontinuation events occurred. axSpA, axial spondyloarthritis; TNFi, tumour necrosis factor inhibitor.

Our survival analysis started with a univariate assessment that identified 5 radiomic features associated with therapy discontinuation (*P* < .25, Supplementary Table S2). Through subsequent multivariate Cox regression analysis after backward selection, we identified 2 shape-based features as independent predictors of TNFi persistence. *Original_shape_Sphericity* increase emerged as a risk factor for discontinuation (hazard ratio [HR] 2.10, 95% CI: 1.09-4.05), while *Original_shape_Elongation* increase showed a protective effect (HR 0.50, 95% CI: 0.25-0.99). Sphericity, which measures the roundness of the BME region relative to a perfect sphere (ranging from 0 to 1), provided insights into the 3-dimensional morphology of the inflammatory lesions; elongation was already previously discussed. Model fit was assessed via Cox-Snell residual analysis, showing reasonable alignment during the observation period when most events occurred (Fig 4).

## DISCUSSION

Our findings suggest that radiomic analysis of SIJ BME may differentiate between axSpA and axPsA with considerable accuracy. The model’s performance, achieving an accuracy of 0.80 and an AUROC of 0.77 using radiomic features alone, without incorporating any clinical parameters, suggests that these conditions may exhibit potentially distinct radiomic phenotypes. The radiomic features that emerged as most significant can be categorised into 3 main groups, each offering different insights into BME characteristics:

1. Shape-based features *(Elongation, MinorAxisLength)* emerged as discriminators, likely reflecting the known differences in sacroiliitis patterns between axSpA and axPsA. The significance of these shape-based features may provide preliminary quantitative support for the previously suggested differences in inflammation patterns, where axSpA typically presents with more symmetric and bilateral involvement, while in axPsA the SIJ involvement often shows asymmetric patterns.
2. Texture features (*NGTDM Coarseness, GLCM IMC1*) emerged also as potential discriminators, suggesting differences in the spatial organisation of BME between the 2 conditions. The significance of these texture parameters is particularly intriguing as they may reflect underlying differences in tissue composition and inflammatory patterns. Recent literature has described distinct immune cell and stromal compositions between enthesis bone and soft tissues, and one may speculate that our texture findings may be related to these underlying biological differences.
3. Grey-level intensity features (LargeAreaEmphasis, LargeAreaHighGrayLevelEmphasis) highlight differences in the distribution and intensity patterns of BME. These features also could be reflecting the distinct nature of inflammation in these conditions as for the previous ones.

The fact that our model could achieve such discrimination using only radiomic features, without clinical parameters, suggests that these features may be capturing fundamental differences between the conditions. This is consistent with recent proposals that axSpA and axPsA could potentially represent distinct pathophysiological entities rather than variants of the same disease [7]. Therefore, radiomic features might be detecting preliminary patterns that reflect different tissue tropisms and inflammatory mechanisms. In this regard, the different underlying genetic factors between these conditions may reflect this ‘splitting’ point of view: *HLA-B27* is the only genetic risk factor common to both diseases, while axial disease in PsA is more frequently associated with other HLA genes than with *HLA-B27* [18,19]*.* Also, baseline serum cytokine levels between axPsA and axSpA differ, with Interleukin (IL)-17A/F baseline serum levels higher in axPsA and lower in axSpA [18]. Histopathology and imaging studies indicate that inflammation in axSpA generally manifests as true bone marrow osteitis centred in the subchondral marrow, whereas in axial PsA inflammation may be more enthesis- or ligament-based (ligamentitis), with less diffuse marrow oedema [20]. Radiographic reviews highlight that patients with axPsA often present asymmetric or unilateral sacroiliitis and less-severe structural damage compared with axSpA, even when axial pain is present [21]. These differences could potentially explain why radiomic shape and texture features may help differentiate axSpA from axPsA.

Our study, therefore, identified preliminary potential differences in radiomic characteristics that might be consistent with the ‘splitters’ theory and thus could reflect different pathophysiological features in the pathology. Because it is not easy to explore the SIJ histologically to date, there is little evidence of histopathological differences between axSpA BME and axPsA BME. With further testing and provided histopathology correlations, radiomics could therefore act as a biopsy surrogate, somewhat like in the field of oncology, where it has indeed proved its worth in functioning as a ‘digital biopsy’, eg, in distinguishing the Programmed Death-Ligand 1 (PD-L1) characteristics of tumour tissues [22], or the BRAF status of melanoma brain metastasis solely from a radiomic perspective [23].

Moreover, while several clinical predictors of TNFi response have been previously identified, our findings suggest that radiomic, particularly shape-based features may provide additional predictive value. The opposing effect of *Sphericity* (HR 2.10, 95% CI: 1.09-4.05) and *Elongation* (HR 0.50, 95% CI: 0.25-0.99) may suggest that the 3-dimensional morphology of BME lesions could potentially reflect underlying disease characteristics relevant to treatment response. More spherical lesions, indicating a more uniform distribution of inflammation, were associated with earlier treatment discontinuation. Conversely, more elongated lesions predicted better treatment persistence.

Currently, the Spondyloarthritis Research Consortium of Canada (SPARCC) scoring system is widely used to quantify SIJ inflammation, and SPARCC baseline scores have shown associations with treatment response [24] to TNFis. In contrast, our radiomic approach does not rely on subjective grading but quantitatively captures 3D lesion shape and texture. In this regard, it remains to be tested whether radiomic features such as sphericity and elongation outperform or complement SPARCC in predicting persistence, especially in early disease stages.

Our preliminary data suggest that noninvasive imaging biomarkers like radiomics might be successfully adapted from oncology [25,26] to inflammatory conditions, improving patient outcomes, as demonstrated in predicting mortality in rheumatoid arthritis associated interstitial lung diseases [27].

Some limitations of our study must be acknowledged. First, our single-centre design and relatively small sample size limit the generalisability of our findings. Despite the use of nested cross-validation and feature selection, the modest sample size (n = 66) and high-dimensional feature set still raise concerns about overfitting in the radiomic model. Even if the cross-validation showed good performance, external independent validation is mandatory to confirm these preliminary findings. This should be addressed in large, multicentre cohorts to achieve reproducibility and generalisability. Specifically, multicentre validation is particularly important given the substantial heterogeneity of MRI imaging protocols (different scanner manufacturers, field strengths, and acquisition parameters) and patient populations (varying demographics, disease characteristics, and clinical practices in applying classification criteria) across different institutions. Also, the radiomic analysis of sacroiliac MRI was cross-sectional, and therefore, the analysis was not conducted on MRI performed at the same disease stage for every patient: this constitutes a limitation that should be taken into account. Additionally, while the majority of patients were biologic-naive, a small subset had prior exposure to TNFis. This heterogeneity in treatment history represents a potential confounding factor, as prior TNFi exposure may influence both imaging characteristics and subsequent treatment response patterns, though the low percentage and similar distribution between groups suggest limited impact on our primary discrimination analysis. Moreover, while our semiautomated segmentation process demonstrated excellent reliability (ICC 0.95), it is not fully automated and therefore may represent a potential source of variability that would need to be addressed in future studies.

Our study focused exclusively on the SIJ BME, despite recognition that (1) structural changes in SIJ are also important in the diagnostic process and that (2) patients with axPsA may also present with isolated spine involvement. This limitation was accepted to facilitate standardisation as structural changes and spinal lesions are not easily accessible with radiomic analysis, and our primary objective was to investigate BME differences between conditions.

For the TNFi persistency analysis, the limited number of discontinuation (n = 10, among 32 patients) represents a critical statistical limitation, underpowering the analysis, even if we accounted for this problem by restricting the number of predictors as discussed in the methods section (sample size justification). These survival analysis results should therefore be interpreted as exploratory and hypothesis-generating.

This study did not include comparison with conventional SPARCC scoring of SIJ inflammation, which remains the semiquantitative standard in clinical practice and prior TNFi response research. Future studies should assess whether radiomic shape and texture features add predictive value over SPARCC when forecasting TNFi persistence.

Above all, future research directions should focus on external validation in larger, multicentre cohorts and the development of standardised imaging and analysis protocols.

## Conclusion

This single-centre, proof-of-concept study suggests that radiomic analysis of SIJ BME shows potential to discriminate between axSpA and axPsA and predict TNFi therapy persistence in patients with axSpA. The ability of our model to achieve discrimination using only radiomic features suggests that these conditions may exhibit distinct radiomic phenotypes. However, these findings should be interpreted as preliminary evidence requiring validation rather than definitive proof of clinical applicability. The modest sample size, single-centre design, and lack of comparison with established scoring systems such as SPARCC represent important limitations that constrain the generalisability of our results. Future research, therefore, should focus on validating these findings in larger, independent, multicentre cohorts and developing standardised protocols for clinical implementation.

## CRediT authorship contribution statement

**Venerito:** Writing – review & editing, Writing – original draft, Validation, Supervision, Software, Methodology, Investigation, Formal analysis, Data curation, Conceptualization. **Sergio Del Vescovo:** Writing – original draft, Visualization, Software, Investigation, Data curation. **Crescenzio Scioscia:** Methodology, Investigation. **Maria Giannotta:** Investigation, Data curation. **Michele De Ceglie:** Software, Methodology, Formal analysis, Data curation. **Antonio Vitale:** Software, Methodology, Investigation, Data curation. **Florenzo Iannone:** Writing – review & editing, Validation, Supervision, Conceptualization. **Giuseppe Lopalco:** Writing – review & editing, Writing – original draft, Supervision, Data curation.

## Appendix Supplementary materials

Image, application 1

Image, video 2 dummy alt text

## Footnotes

Supplementary material associated with this article can be found in the online version at doi:10.1016/j.ero.2025.12.007.
