# Supplementary material for: Radiomics to discriminate between axial spondyloarthritis and axial psoriatic arthritis and to predict TNFi therapy persistence
Source: EULAR Rheumatol Open. 2025 Dec 27;2(1):50–7. doi: 10.1016/j.ero.2025.12.007 (PMC13292495; doi:10.1016/j.ero.2025.12.007)
Supplement: Supplementary file 1 [file mmc1.docx]

**SUPPLEMENTARY MATERIALS**

**Description of the 120 standardized radiomic features across seven distinct feature classes**

- **First-order Statistics (19 features)**: These features describe the distribution of voxel intensities within the BME region without considering spatial relationships. They include fundamental statistical measures such as mean intensity, standard deviation, skewness (asymmetry of intensity distribution), kurtosis (peakedness of distribution), energy (sum of squared intensities), entropy (randomness of intensity distribution), and various percentile measurements. These features reflect overall signal intensity characteristics and heterogeneity of the bone marrow edema.
- **Shape-based 3D Features (16 features)**: These features quantify the three-dimensional morphological properties of the segmented BME regions. Key measurements include volume, surface area, sphericity (how closely the shape resembles a perfect sphere), compactness (relationship between volume and surface area), maximum 3D diameter, elongation (ratio of principal component axes), flatness (deviation from spherical shape), and surface-to-volume ratio. Shape features provide insights into the geometric patterns of inflammatory involvement in sacroiliac joints.
- **Gray Level Co-occurrence Matrix Features (GLCM, 24 features)**: GLCM features analyze spatial relationships between adjacent voxels by examining how frequently different intensity combinations occur at specified distances and directions. These include contrast (local intensity variation), correlation (linear dependency between neighboring intensities), energy (uniformity of intensity pairs), homogeneity (closeness of intensity distribution), dissimilarity (difference between neighboring intensities), and various entropy measures. GLCM features capture fine-scale texture patterns that may reflect underlying tissue microstructure and inflammatory organization.
- **Gray Level Run Length Matrix Features (GLRLM, 16 features)**: GLRLM features characterize texture by analyzing consecutive voxels with identical gray levels in specific directions. Key features include short run emphasis (prevalence of short uniform runs), long run emphasis (prevalence of long uniform runs), gray level non-uniformity (variation in gray level runs), run length non-uniformity (variation in run lengths), and run percentage (proportion of runs to total voxels). These features detect directional texture patterns that may indicate organized versus chaotic inflammatory processes.
- **Gray Level Size Zone Matrix Features (GLSZM, 16 features)**: GLSZM features quantify regions of connected voxels with identical gray levels, regardless of shape. Important measurements include small area emphasis (prevalence of small uniform zones), large area emphasis (prevalence of large uniform zones), gray level non-uniformity (variation in gray levels across zones), size zone non-uniformity (variation in zone sizes), and zone percentage (proportion of zones to total voxels). These features capture regional homogeneity patterns within the BME that may reflect different stages or types of inflammatory activity.
- **Gray Level Dependence Matrix Features (GLDM, 14 features):** GLDM features measure dependencies between each voxel and its neighborhood, providing information about local texture organization. Key features include dependence non-uniformity (variation in dependence counts), gray level non-uniformity (variation in gray levels), dependence entropy (randomness of dependence patterns), and dependence percentage (proportion of dependent voxels). These features capture local neighborhood relationships that may reflect tissue organization patterns.
- **Neighboring Gray Tone Difference Matrix Features (NGTDM, 5 features)**: NGTDM features analyze differences between each voxel and its neighboring voxels, providing measures of local texture complexity. These include coarseness (average difference between neighboring intensities), contrast (difference between highest and lowest intensities), busyness (spatial frequency of intensity changes), complexity (information content of texture), and strength (primitives visibility). NGTDM features quantify the spatial rate of intensity change, which may reflect the sharpness or gradual nature of inflammatory boundaries.

| **Hyper-parameter** | **Grid values tested** | **Best value (median)** |
| --- | --- | --- |
| n_estimators | 100, 200, 300, 400, 500 | **174** |
| learning_rate | 0.01, 0.05, 0.10, 0.20 | **0.05** |
| max_depth | 3, 4, 5, 6 | **4** |
| min_child_weight | 1, 2, 4 | **2** |
| subsample | 0.60, 0.80, 1.00 | **0.80** |
| colsample_bytree | 0.60, 0.80, 1.00 | **0.80** |
| gamma | 0, 0.10, 0.30 | **0.00** |
| lambda | 1, 5, 10 | **5** |

**Supplementary Table S1.** XGBoost grid-search space and winning values (median across the five outer CV folds)

| **Variable Name** | **HR (exp coef)** | **95% exp coef lower** | **95% exp coef upper** | **p** |
| --- | --- | --- | --- | --- |
| diagnostics_Image-original_Mean | 1.00219 | 0.999023 | 1.00536 | 0.175733 |
| diagnostics_Mask-original_VolumeNum | 0.937821 | 0.844898 | 1.04096 | 0.227874 |
| original_shape_Elongation | 0.0857516 | 0.00243509 | 3.01974 | 0.176452 |
| original_shape_Flatness | 0.0403536 | 0.00019107 | 8.5226 | 0.239838 |
| original_shape_Sphericity | 89.3718 | 0.491412 | 16253.8 | 0.0905803 |

**Supplementary Table S2**. Cox Proportional Hazards Analysis Results for Radiomic Features. HR = Hazard Ratio; exp coef lower/upper = 95% confidence interval bounds for the hazard ratio; p = p-value. Analysis includes diagnostic image parameters, mask volume measurements, and original shape characteristics (elongation, flatness, and sphericity). None of the features demonstrated statistically significant associations at α = 0.05.


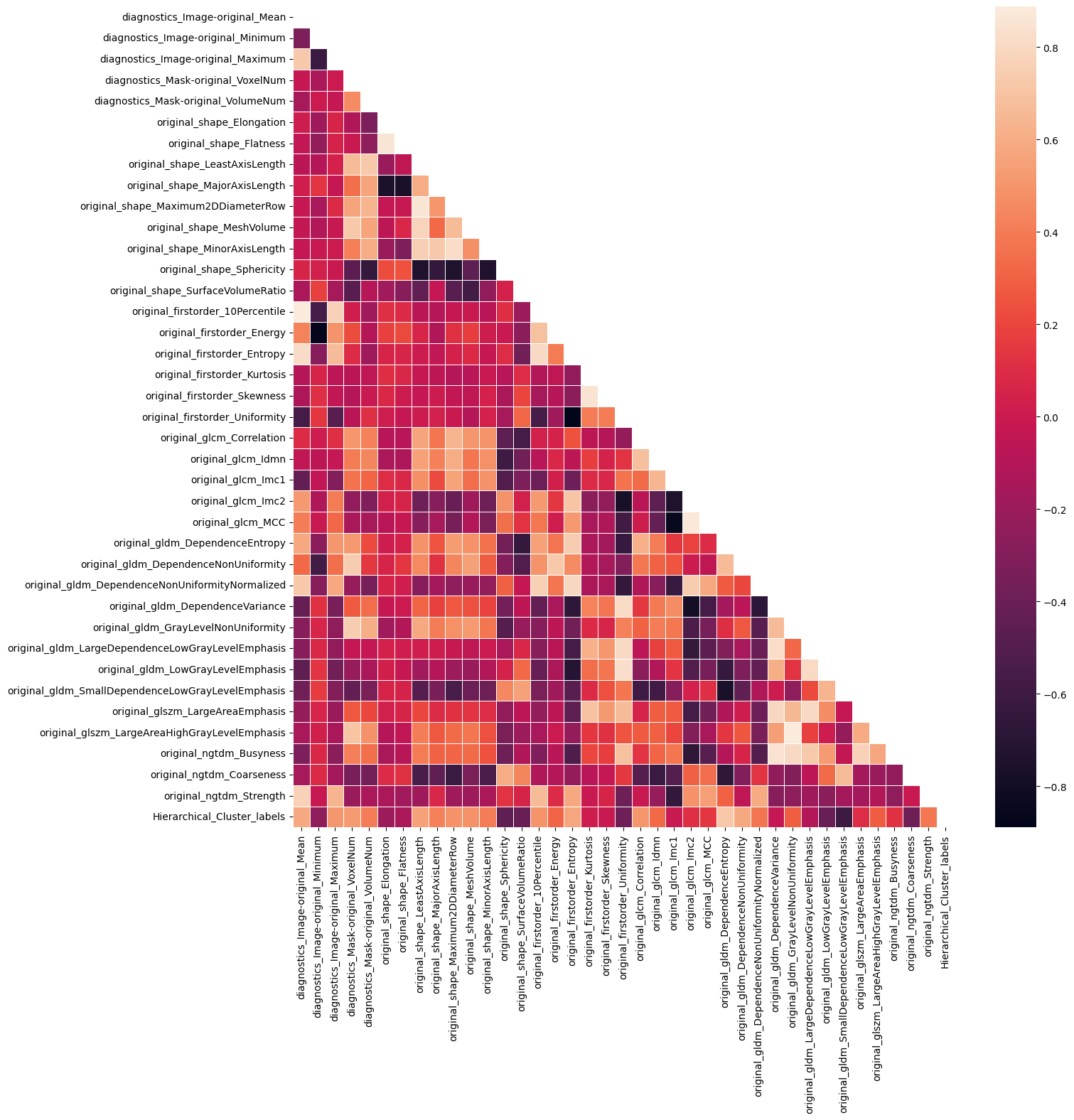


**Supplementary Figure 1.** Correlation heatmap of the 39 retained radiomic features after feature selection. The heatmap displays Pearson correlation coefficients between all pairs of features, with color intensity representing correlation strength (scale: -0.8 to +0.8). Features include diagnostic parameters (n=5), original shape characteristics (n=9), first-order statistics (n=6), Gray Level Co-occurrence Matrix (GLCM) features (n=5), Gray Level Dependence Matrix (GLDM) features (n=8), Gray Level Size Zone Matrix (GLSZM) features (n=2), Neighboring Gray Tone Difference Matrix (NGTDM) features (n=3), and hierarchical cluster labels (n=1). Lighter colors indicate positive correlations, darker colors indicate negative correlations, and intermediate colors represent weak or no correlation
